# Susceptibility of *Porphyromonas gingivalis* and *Streptococcus mutans* to Antibacterial Effect from *Mammea americana*

**DOI:** 10.1155/2014/384815

**Published:** 2014-04-24

**Authors:** Alejandra Herrera Herrera, Luis Franco Ospina, Luis Fang, Antonio Díaz Caballero

**Affiliations:** ^1^Grupo de Investigaciones GITOUC, Facultad de Odontología, Universidad de Cartagena, Campus de la Salud, Cartagena, Colombia; ^2^Grupo de Evaluación Biológica de Sustancias Promisorias, Facultad de Ciencias Farmacéuticas, Universidad de Cartagena, Campus de la Salud, Cartagena, Colombia

## Abstract

The development of periodontal disease and dental caries is influenced by several factors, such as microorganisms of bacterial biofilm or commensal bacteria in the mouth. These microorganisms trigger inflammatory and immune responses in the host. Currently, medicinal plants are treatment options for these oral diseases. *Mammea americana* extracts have reported antimicrobial effects against several microorganisms. Nevertheless, this effect is unknown against oral bacteria. Therefore, the aim of this study was to evaluate the antibacterial effect of *M. americana* extract against *Porphyromonas gingivalis* and *Streptococcus mutans*. For this, an experimental study was conducted. Ethanolic extract was obtained from seeds of *M. americana* (one oil phase and one ethanolic phase). The strains of *Porphyromonas gingivalis* ATCC 33277 and *Streptococcus mutans* ATCC 25175 were exposed to this extract to evaluate its antibacterial effect. Antibacterial activity was observed with the two phases of *M. americana* extract on *P. gingivalis* and *S. mutans* with lower MICs (minimum inhibitory concentration). Also, bactericidal and bacteriostatic activity was detected against *S. mutans*, depending on the concentration of the extract, while on *M. americana* extract presented only bacteriostatic activity against *P. gingivalis*. These findings provide important and promising information allowing for further exploration in the future.

## 1. Introduction

Oral diseases are a worldwide public health problem. Many epidemiological studies report that diseases such as dental caries and periodontal disease are the most prevalent oral disorders of humanity [1–3]. These conditions are caused by poor oral hygiene and biofilm forming bacteria residing in the mouth, able to communicate with each other through mechanisms of Quorum sensing [4]. Biofilms are complex structures, where different bacterial species are arranged to form a superorganism with advanced properties unlike planktonic bacteria. Dental or bacterial plaque is a type of biofilm on the tooth surface that plays an important role in the development of these oral conditions [5, 6].* Streptococcus mutans* colonize the tooth surface and initiate biofilm formation by their ability to synthesize extracellular polysaccharides from sucrose [7]. The further accumulation of biofilm around the supra- and subgingival region leads to a shift in its microbial composition from* Streptococcus *spp.,* Actinomyces *spp., and* Porphyromonas gingivalis* [8, 9]. Therefore, these microorganisms are considered to be the major etiological agents involved in dental caries and periodontal disease [3].

Clinical practice seeks to prevent the occurrences of these oral conditions or apply minimally invasive treatments, avoiding in most cases surgical interventions [10, 11]. Therefore, antimicrobial or antibacterial agents against these oral pathogens could play an important role in the prevention and treatment of dental caries and periodontal disease, principally those that can inhibit or reduce the growth of these microorganisms, inhibit biofilm formation, influence the adhesion of bacteria to surfaces, and reduce the clinic symptoms [12, 13]. Many of the currently available oral antimicrobials can change oral microbiota and have adverse side effects such as diarrhea, vomiting, and teeth staining. Therefore, it is necessary to find safe and effective therapeutic agents for everyone. Traditional herbal medicines are considered as a good source of therapeutic alternatives, depending on their properties [14, 15]. Antimicrobial agents isolated from plants represent a huge and poorly exploited source of drugs with great therapeutic potential. Some of these compounds are effective in the treatment of infectious diseases, while having the advantage of causing few side effects which are often associated with synthetic antimicrobials [16].

*Mammea americana* is an evergreen tree of the family Calophyllaceae, native of the Antilles, and introduced into central and northern South America, although it is currently cultivated in other tropical and humid areas. It is commonly known as mammee, mammee apple, mamey, mamey apple, Santo Domingo apricot, South American apricot, mamey de cartagena de indias, or mamey de santo domingo. Its fleshy fruit is edible and frequently consumed; they have a yellow-reddish and aromatic pulp and are round or slightly irregular, with a thick brown rind. Their diameter ranges from 10 to 20 cm. Small fruit contain a single seed, while larger ones might have up to four. The seeds are brown, rough, oval, and around 6 cm long [17].

Oral lore has referred the use of* M. americana* as a natural therapeutic alternative. However, the medicinal properties of the leaves, fruits, and seeds have not been widely elucidated. Several studies have reported some medicinal properties, such as anticonvulsant, antipyretic, antimalarial, anthelmintic, and digestive tonic, as well as a remedy for parasitic skin diseases [18]. It also provides inhibitory activity against* Mycobacterium tuberculosis* [19] and molluscicidal properties against* Biomphalaria glabrata* [20]. Furthermore, antitumor activity has been reported, as well as some coumarin, phloroglucinol derivatives [21, 22], xanthones, and benzophenones [23]. Currently, there are few studies reporting the* M. americana* antimicrobial activity, specifically for oral bacteria. Therefore, the aim of this study was to identify the mamey's antibacterial activity against pathogenic bacteria involved in dental caries and periodontal disease.

## 2. Materials and Methods

### 2.1. Plant Material

*Mammea americana* fruit was collected from rural areas in the Department of Bolivar on the Caribbean coast of Colombia. Geographical and environmental conditions in this tropical zone promote the wild growth of* M. americana*. Voucher specimens were prepared and identified at the Universidad de Antioquia Herbarium (*HUA 183928*).

### 2.2. Extract Preparation

From the ripe fruit of* M. americana*, we extracted the seeds. These were air dried at 25°C for 3 weeks and ground in a seed grinder into a fine powder. The powdered materials (1313 g) were soaked in ethanol (50% w/v) at room temperature for 72 h in dark conditions and then were filtered. The total extract was dried, and solvent was evaporated in a rotary evaporator (*Laborota 4001, Heidolph*) under reduced pressure at 50°C. We obtained two phases from the total extract: one hydroalcoholic phase called “ethanolic phase” and one “oily phase,” 90 g and 109 g, respectively. Both phases were further dried at room temperature; before evaluating the antibacterial activity, each phase was subjected to a solubility test in 2% ethanol, 2% methanol, 1% DMSO (dimethyl sulfoxide), and combinations from these solvents. We observed that 1% DMSO was the best solvent for both phases. In addition, 1% DMSO did not exert any damage on the bacterial strains.

### 2.3. Preliminary Phytochemical Screening

Several chemical tests were carried out on two phases of the* M. americana* extract using procedures to identify the following groups of metabolites: flavonoids and xanthone (Shinoda test and Action front of álcalis), leucoanthocyanidins (Rosenheim test, NaOH 10% test, and hydrochloric acid test), phenolic compounds (FeCl_3_ test), quinones (RX with sulfuric acid), cardiotonic glycoside (Kedde test), steroid nucleus (Salkowski test), alkaloids (Dragendorff test, Mayer test, Wagner test, and FeCl_3_ test), tannins (FeCl_3_ test), saponins (Foam test), and coumarins (coumarins volatile test, ammonium hydroxide Rx) [24].

### 2.4. Microorganisms and Growth Conditions

This study only included* Porphyromonas gingivalis* (ATCC 33277) and* Streptococcus mutans* (ATCC 25175), both acquired from the American Type Culture Collection.* P. gingivalis* was grown in Brucella agar (BD,* Becton Dickinson*), supplemented with vitamin k1 (1 mg/mL) - hemin (5 *μ*g/mL) solution (BD,* Becton Dickinson*) and 5% human anticoagulated whole blood, and incubated at 37°C under anaerobic conditions in an anaerobic jar with AnaeroGen (90% N_2_, 5% CO_2_, and 5% H_2_) (Oxoid Ltd.) for 5 days, while* S. mutans* was cultured in TYS20B agar. This culture medium contains 30 g trypticase soy (BD,* Becton Dickinson*), 10 g yeast extract (Oxoid Ltd.), 20% w/v sucrose (Merck), 0.2 U/mL bacitracin (Sigma-Aldrich), 11 g granulated agar, and distilled water and incubated at 37°C under anaerobiosis by AnaeroGen (Oxoid Ltd.) for 48 h.

From a few microbial colonies of* P. gingivalis* and* S. mutans* bacterial cultures, inocula and bacterial growth curves were performed for each strain. Isolated colonies were suspended in their corresponding culture media and turbidity of the inoculum was adjusted to reach 0.5 on the McFarland scale (optical density between 0.08 and 0.10) using a microplate reader (*Multiscan EX, Thermo Scientific*) at 620 nm, which is equivalent to 1–2 × 10^8^ CFU/mL.* S. mutans* reached its stationary phase for approximately 13 h, whereas* P. gingivalis* required 22 h of incubation. These preliminary tests allowed establishing ideal experimental conditions.

### 2.5. Antibacterial Activity Assay

Antibacterial activity was determined by the microdilution technique with 96-well microplates. Using this technique, minimum inhibitory concentration (MIC) and minimum bactericidal concentration (MBC) values were obtained for the ethanolic phase and oily phase against the microorganisms under study. All assays were performed in triplicate.

#### 2.5.1. Determination of the Minimum Inhibitory Concentration (MIC)

The ethanolic and oily phases were serially diluted, ranging from 500 *μ*g/mL to 0.06 *μ*g/mL in the 96-well plate with bacterial suspension (5 × 10^5^ CFU/mL); gentamicin (16 *μ*g/mL) was used as negative control for bacterial growth, and a broth solution was used as control sterility. Elsewhere, microbial growth was indicated as changes in optical density from bacterial inoculum (positive control). The 96-well plates were incubated at 37°C under anaerobic conditions, for 13 h (*S. mutans*) and 22 h (*P. gingivalis*). The MIC was defined as the lowest concentration that inhibited microbial growth.

#### 2.5.2. Determination of the Minimum Bactericidal Concentration (MBC)

The MBC was determined by adding 10 *μ*L of the suspensions from the wells, which did not show any growth during MIC assays in petri dishes with corresponding agar to each bacterium. These petri dishes were incubated at 37°C under anaerobic conditions for 5 days (*P. gingivalis*) and for 48 h (*S. mutans*). After this incubation period, bacterial colonies were observed. The ethanolic and oily phases were designated as bacteriostatic (those in which bacterial colonies grew in petri dishes) or bactericide (those in which bacterial colonies did not grow in petri dishes).

### 2.6. Statistical Analysis

The data were analyzed using GraphPad Prism v5 and compared by nonparametric Kruskal-Wallis and multiple-comparison tests as the Dunnett's test; this was applied for comparison between each treatment concentration and the respective control. The chosen level of significance for all statistical tests was *P* < 0.05.

## 3. Results

### 3.1. Antibacterial Activity

**Figure 1:**
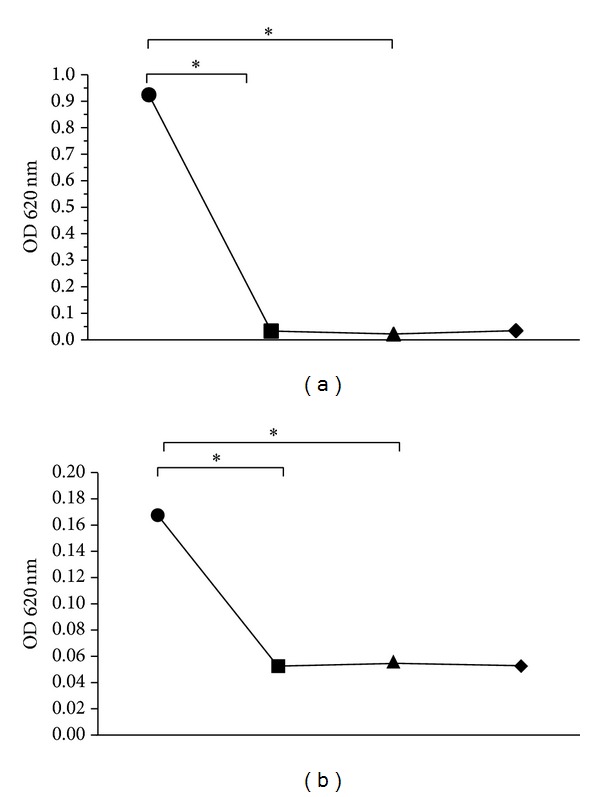
Bacterial sensitivity assays of* Mammea americana* extract. (a) Sensitivity of* Porphyromonas gingivalis* (ATCC 33277) to oily and ethanolic phases; exposure of* P. gingivalis* to mamey extract inhibited bacterial growth by approximately 96%. (b) Sensitivity of* Streptococcus mutans* (ATCC 25175) to mamey extract; the two phases inhibited bacterial growth by approximately 31.5%. Each symbol represents the mean for the group (*n* = 6 per group); inoculum: (●), oily phase (500 *μ*g/mL): (■), ethanolic phase (500 *μ*g/mL): (▲), control (gentamicin (16 *μ*g/mL)): (◆), and *P* value < 0.05: (∗); Kruskal-Wallis test and Dunnett's posttest.

The two phases of* M. americana* extract proved antibacterial activity against* S. mutans* and* P. gingivalis* strains. These bacteria were significantly sensitive to the extract from 500 *μ*g/mL, from a mean optical density (OD) of 0.93 for the inoculums of* P. gigivalis* to 0.033 (*P* = 0.005) and 0.022 (*P* = 0.005) for wells with bacteria exposed to the oily and ethanolic phases, respectively. These results are similar and do not differ from those of gentamicin OD: 0.034, (*P* = 0.32); therefore, mamey extract inhibits bacterial growth by 96% (Figure 1(a)).* S. mutans* was susceptible to mamey and inhibits bacterial growth by approximately 31.5%, from a mean OD of 0.170 for the inoculums of* S. mutans* to 0.053 (*P* = 0.013) for oily phase and 0.055 (*P* = 0.013) for ethanolic phase (Figure 1(b)).

**Figure 2:**
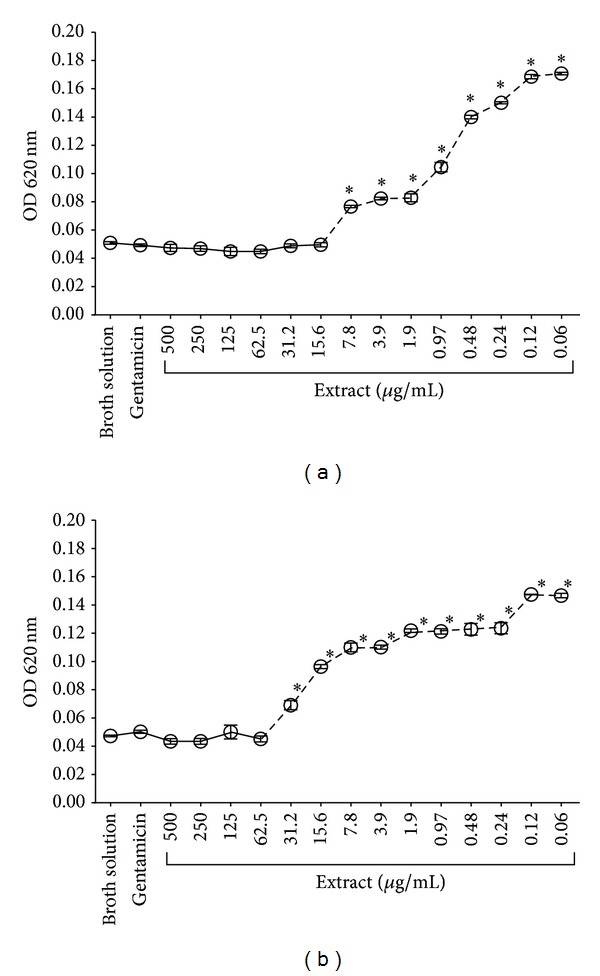
MIC of the oily and ethanolic phases from* Mammea americana* extract against* Streptococcus mutans*. (a) The minimum concentration of the oily phase which inhibited bacterial growth was 15.6 *μ*g/mL, (b) whereas the MIC of ethanolic phase was 62.5 *μ*g/mL. Each symbol represents the mean ± SEM for each concentration tested (*n* = 4 per (*μ*g/mL)). *P* value < 0.05: (∗); Kruskal-Wallis test and Dunnett's posttest.

**Figure 3:**
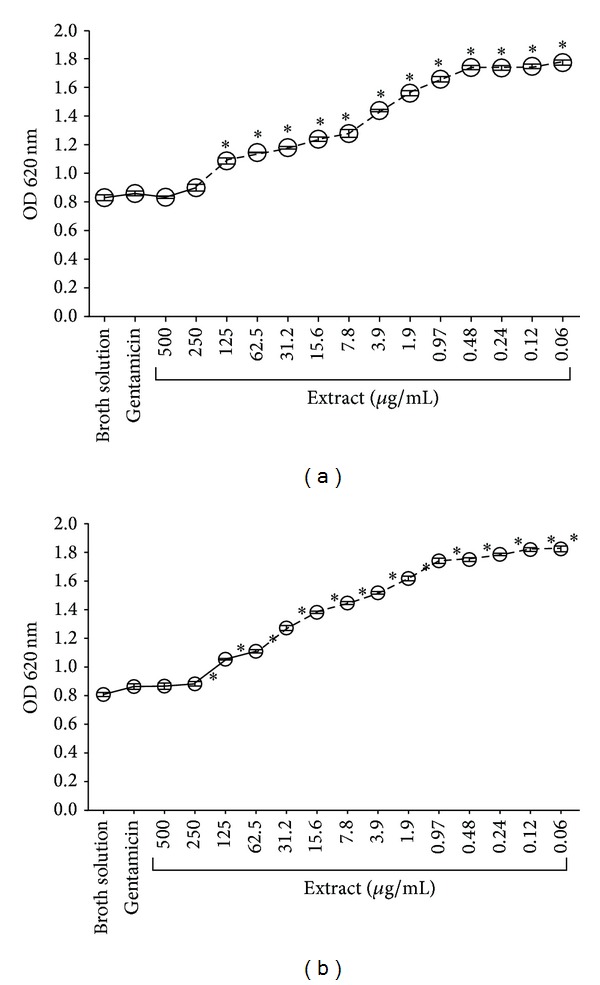
MIC of the oily and ethanolic phases from* Mammea americana* extract against* Porphyromonas gingivalis*. (a) The MIC of the oily phase was 250 *μ*g/mL, (b) while the MIC of the ethanolic phase was 500 *μ*g/mL. Each symbol represents the mean ± SEM for each concentration tested (*n* = 4 per (*μ*g/mL)). *P* value < 0.05: (∗); Kruskal-Wallis test and Dunnett's post-test.

This antibacterial activity was interpreted as minimum inhibitory concentration (MIC) and minimum bactericidal concentration (MBC). The oily and ethanolic phases showed very promising data on Gram-positive bacteria, which was reflected by their MICs against* S. mutans* (MIC: 15.62 *μ*g/mL and 62.5 *μ*g/mL, resp.), comparing broth solution at all tested concentrations (Figures 2(a) and 2(b)). While the two phases did not show prominent results against* P. gingivalis*, their MICs showed antibacterial activity; the MIC of the oily phase was 250 *μ*g/mL and 500 *μ*g/mL for the ethanolic phase (Figures 3(a) and 3(b)).

MBC was determined from 500 *μ*g/mL of each phase. The oily phase showed bactericidal property against* S. mutans* from 500 *μ*g/mL to 125 *μ*g/mL, and concentrations <125 *μ*g/mL behaved as bacteriostatic. While the ethanolic phase showed bactericidal activity from 500 *μ*g/mL to 250 *μ*g/mL, lower concentrations were bacteriostatic.* M. americana* extract did not show bactericidal activity against* P. gingivalis*. Both oily and ethanolic phases only showed bacteriostatic behavior even at 500 *μ*g/mL (Table 1).

### 3.2. Phytochemical Screening

The phytochemical screening of the oily and ethanolic phases showed the presence of phenolic compounds, tannins, and coumarins (Table 2). These metabolites possibly contribute to the antibacterial activity of* M. americana* extract.

## 4. Discussion

Oral bacteria have been amply tested for antimicrobial susceptibility to various plant extracts and natural substances. According to our knowledge, this is the first report on the antibacterial effect of* M. americana* extract against bacteria representative of oral diseases such as dental caries and periodontal disease such as* S. mutans* and* P. gingivalis* [8, 9].

*M. americana*, commonly known as “mamey,” is a tree widely used for its medicinal properties and its fruit is sought in communities of the Antilles and central and northern South America. Morris and Pagán. were the first to report some properties of mamey, including antimicrobial and toxic properties of fruit seeds of this tree [25]; other parts of mamey, such as leaves, stems, and fruit, have also shown medicinal properties. Frame et al. evaluated the antituberculous effect against* Mycobacterium smegmatis* from 50 extracts of plants belonging to tropical flora in Puerto Rico. The* Mammea americana* extract showed the best antituberculous effect with low concentration (50 *μ*g) unlike 49 remaining extracts whose concentrations are close at 500 *μ*g, this showed promising properties for* M. americana* [19]. We also observe similar promising results of fruit seeds of mamey against oral bacteria.

For a better knowledge, there are no papers reporting antimicrobial activity of* Mammea americana* extract tested on bacteria such as* Streptococcus mutans* and* Porphyromonas gingivalis*. However, other plant extracts were tested on the same bacteria; for instance, Fani and Kohanteb, in 2012 [26], assessed Aloe Vera against* Streptococcus mutans* and they founded a MIC with 25 ppm against that bacterium. Iauk et al., in 2003 [27], reported that extract of* Hamamelis virginiana* had a MIC of 512 ppm against* Porphyromonas gingivalis*. Regarding the MBC Moreno et al. in 2007 [28] reported that 460 ppm of* propolis* was a MBC against* Streptococcus mutans*. Bakri and Douglas in 2005 [29] observed a MBC of* Allium sativum* (garlic) against* Porphyromonas gingivalis* 7.9 mg/mL. Comparing these results with the activity of the extract of* Mammea americana* proposed in this paper, it can be noted that present results of* Mammea americana* have a higher potential or more promising behavior such as medicament for the observed outcome of inhibitory activity against these oral bacteria.

Due to the fatty nature of the mamey fruit seeds, a total extract composed of two phases was obtained, which evaluated their antibacterial activity. Few studies have reported this property; Yasunaka et al. evaluate the antibacterial activity on* Escherichia coli* and* Staphylococcus aureus* of 32 crude extracts from 22 Mexican medicinal plants. Some extracts evaluated showed antibacterial activity in one of two bacterial strains, but only the leaves extract of* M. americana* inhibited bacterial growth of the two strains tested [30]. We also obtained similar results, considering that this property was assessed on* S. mutans* and* P. gingivalis*.

It remains to elucidate the active compounds and cytotoxic effect of* M. americana* seeds extract. However, some metabolites present in mamey have reported antimicrobial activity for other medicinal plants. The major metabolite of mamey reported in other studies is coumarin [31], including* Mammea africana*, another species of the Guttiferae family; coumarins of this species exhibit significant antimicrobial activity against* S. aureus* [32]. Other metabolites found in mamey are the phenolic compounds, the antimicrobial capacity of these, acting by the alteration of the permeability of the cell membrane that could result in the uncoupling of oxidative phosphorylation, inhibition of active transport, and loss of pool metabolites due to cytoplasmic membrane from bacteria [33, 34]. These could be two possible explanations for the antibacterial activity of the* M. americana* seeds extract reported in our study.

In conclusion, we may point out that the* Mammea americana* extract showed antibacterial activity against* Porphyromonas gingivalis* ATCC 33277 and* Streptococcus mutans* ATCC 25175, and the MIC-MBC obtained in this work allows us to hypothesize that* M. americana* presents promising effects against oral bacteria which need to be further studied.

## Figures and Tables

**Table 1:** Minimum bactericidal concentration MBC from the oily and ethanolic phases against *Streptococcus mutans *and *Porphyromonas gingivalis *strains.

Bacterial strain	Phase	Results
*Streptococcus mutans *	Oily	Bactericidal activity from 500–125 µg/mL; lower concentrations were bacteriostatic.
	Ethanolic	Bactericidal activity from 500–250 *µ*g/mL; lower concentrations were bacteriostatic.

*Porphyromonas gingivalis *	Oily	It showed bacteriostatic activity at all concentrations.
	Ethanolic	It showed bacteriostatic activity at all concentrations.

**Table 2:** Phytochemical screening of the oily and ethanolic phases.

Chemical components	Oily phase	Ethanolic phase
Flavonoides (xanthone and flavone)	−	+
Leucoanthocyanidins	+	−
Phenolic compounds	+++	+++
Quinones	−	−
Cardiac glycosides	−	−
Steroid nucleus	−	+
Alkaloids	−	−
Coumarins	+++	+++
Tannins	++	+++
Saponins	−	−

Absent: −; present: + (mild), ++ (moderate), and +++ (severe).
